# Bioabsorbable magnesium screw versus conventional titanium screw fixation for medial malleolar fractures

**DOI:** 10.1186/s10195-020-00547-7

**Published:** 2020-05-25

**Authors:** Hasan May, Yusuf Alper Kati, Gurkan Gumussuyu, Tuluhan Yunus Emre, Melih Unal, Ozkan Kose

**Affiliations:** 1 Department of Orthopedics and Traumatology, Antalya Training and Research Hospital, Antalya Egitim ve Arastirma Hastanesi, Soğuksu mah. Kazım Karabekir cd. Muratpaşa, 07100 Antalya, Turkey; 2 Department of Orthopedics and Traumatology, Medical Faculty, Biruni University, Istanbul, Turkey; 3 Department of Orthopedics and Traumatology, Medical Faculty, Altınbaş University, Istanbul, Turkey

**Keywords:** Ankle fracture, Medial malleolar fracture, Implant removal, Magnesium screw, Titanium screw, Bioabsorbable implant

## Abstract

**Background:**

It is still unknown whether bioabsorbable magnesium (Mg) screws provide an advantage over titanium screws in the treatment of medial malleolar (MM) fractures. The purpose of this retrospective study is to compare the clinical and radiological outcomes of MM fractures fixed with either bioabsorbable Mg screws or conventional titanium screws.

**Materials and methods:**

A cohort of 48 patients with MM fractures who underwent compression screw fixation was retrospectively reviewed. Twenty-three patients (16 male, 7 female; mean age: 37.9 ± 17.7 years) were treated with bioabsorbable Mg screws, and 25 patients (14 male, 11 female; mean age: 45.0 ± 15.7 years) were treated with conventional titanium screw fixation. All patients were followed up for at least 1 year, with a mean time of 24.6 ± 10.5 months (12–53 months). The American Orthopedic Foot and Ankle Society (AOFAS) scale was used to evaluate the clinical results. The Kellgren–Lawrence (KL) osteoarthritis grading was used to evaluate posttraumatic osteoarthritis on final ankle radiographs. Fracture union, rate of implant removal, and complications were recorded. Comparative analysis of two independent groups was performed using the chi-squared test and the Mann–Whitney *U*-test.

**Results:**

The two groups were comparable concerning demographic and clinical characteristics. Age (*p* = 0.146), sex (*p* = 0.252), side (*p* = 0.190), MM fracture type (*p* = 0.500), associated fractures (*p* = 0.470), and follow-up period (*p* = 0.903) were similar between the groups. At final follow-up examination, AOFAS score (*p* = 0.191) was similar between groups. Fracture union was achieved in all cases. Grade of posttraumatic osteoarthritis, according to KL, was equally distributed in both groups (*p* = 0.074). No deep infection or osteomyelitis was seen. Five patients in the titanium screw group underwent implant removal, due to pain in three of them and difficulty in wearing shoes in the other two (*p* = 0.031). Implant removal was performed after a mean of 14.2 ± 3.1 months (12–19 months).

**Conclusions:**

Bioabsorbable Mg and titanium screws had similar therapeutic efficacy in MM fracture fixation regarding functional and radiological outcomes. However, the rate of implant removal was higher with titanium screws. Bioabsorbable Mg screws may be a favorable fixation option since secondary implant removal procedures can be prevented.

**Level of evidence:**

Level IV, Retrospective case series.

## Introduction

Ankle fractures are some of the most common musculoskeletal injuries in adults and constitute 9% of all fractures [1]. In a large population-based study spanning a decade, the incidence of ankle fractures was reported to be 168.7/100,000 persons/year [2]. Given that ankle fractures are common injuries, the indications for conservative and surgical treatment are well described, and widely accepted management strategies are currently available. Isolated stable medial malleolar (MM) fractures with less than 2 mm displacement can be treated conservatively with cast immobilization. However, in unstable ankle fractures such as bimalleolar and trimalleolar fractures, surgical fixation of all fractures is usually advocated to restore ankle stability [3, 4]. Although numerous implants and techniques have been described and used to fix MM fractures, transverse and oblique fractures are usually secured with two compression screws, and buttress plate fixation is recommended for vertical fractures [4]. Several previous studies reported satisfactory functional and radiological results following MM fractures. Regardless of the fixation technique and implant used, anatomical reduction of the fracture and preservation of the reduction during fracture union has been shown to be the most critical factor affecting the outcome [4, 5].

Implant removal after fixation with titanium or steel implants is a significant clinical problem in orthopedic surgery. The ankle is the most common anatomic site where implant removal operations are performed [6, 7]. This second surgery increases overall costs of the treatment, may cause complications, and may also create a psychological burden for the patient [6–9]. The production of bioabsorbable implants and their introduction into orthopedic surgery represents important progress to eliminate this problem. Most of the bioabsorbable implants used today are made of poly-l-lactide (PLA), polyglycolide (PGA), polydioxanone (PDO), or their copolymers and derivatives [10]. However, several undesirable reactions and complications, including osteolysis, foreign body reaction, and sinus formation, have been observed with these implants [10, 11]. To overcome these problems, implants made from elements such as magnesium (Mg) that are naturally present in the body and are involved in the structure of the bone and the bone metabolism have been developed. Mg and its alloys have unique properties that make them favorable biomaterials for manufacturing orthopedic implants. The density (1.74 g/cm^3^) and the elastic modulus (41–45 GPa) of Mg are very close to cortical bone (2.1 g/cm^3^ and 10–40 GPa, respectively) [12]. Moreover, the degradation products are not toxic and may even induce or promote new bone formation and provide resistance to infection [13–15]. Among the various Mg alloys, magnesium, yttrium, rare earth element, and zirconium (MgYREZr) implants entered the market in 2013 after being clinically approved and obtaining the CE mark, and are still in use [16].

Promising clinical results have been reported with bioabsorbable Mg implants in various indications, including ankle fractures [16–19]. However, only one previous study reported the short-term clinical outcomes of bioabsorbable Mg screw fixation of MM fractures in a limited number of patients without a comparison with conventional implants [18]. The present study aims to compare bioabsorbable Mg and conventional titanium screw fixation in MM fractures.

## Materials and methods

### Patients and study design

A retrospective review was performed on all patients with ankle fractures who underwent operative treatment in our institution between January 2015 and January 2018. All the radiologic imaging files which were stored in a picture archiving and communication system (PACS), patients’ charts, medical records, operation notes, and notes taken during the follow-up visits were obtained from the institutional patient database and used to extract the demographic information, clinical findings, and imaging findings. Among these patients, those with MM fractures (either isolated or accompanying bimalleolar or trimalleolar ankle fractures) fixed with two compression screws were selected. Skeletally immature patients with open physis, fixation implants other than compression screws such as plates or tension band technique, and patients with follow-up shorter than 12 months were excluded from the study. After application of the inclusion and exclusion criteria as mentioned above, 48 eligible patients who underwent MM fracture fixation with two compression screws were identified and included in this study. Twenty-five of these patients were treated with two titanium compression screws (4.0 mm or 4.5 mm partial thread cannulated screws) (Group Ti), and the remaining 23 were treated with bioabsorbable Mg headless compression screws (3.2 mm, MAGNEZIX^®^ CS; Syntellix AG, Hanover, Germany) (Group Mg). This study was carried out following the ethical standards laid down in the 1964 Declaration of Helsinki and its later amendments, and the institutional review board (IRB) approved the study protocol (IRB approval date/issue: 2019/375.27/4).

### Clinical and radiological assessments

MM fractures were classified using the Herscovici classification system [3]. Anteroposterior and lateral ankle radiographs taken at final follow-up were used for the evaluation of fracture union, malunion, and degree of posttraumatic ankle osteoarthritis. Clinical outcomes were assessed with the American Orthopaedic Foot and Ankle Society (AOFAS) Ankle-Hindfoot scale [20]. Any complications, including infection, wound problems, implant failure, ankle instability, and tendon subluxation or synovitis, during follow-up were recorded. Time of implant removal (if it was performed) and the reason for removal were recorded. The Kellgren–Lawrence (KL) osteoarthritis grading was used to evaluate posttraumatic osteoarthritis on final ankle radiographs [21]. All available serial follow-up radiographs and computed tomography (CT) scans (if available) were evaluated to monitor the degradation of bioabsorbable Mg screws.

### Statistical analysis

Continuous variables are presented as mean ± standard deviation (SD), median, and range. Categorical variables are stated as percentages and frequency distribution. The Kolmogorov–Smirnov test was used to determine whether the data were distributed normally. A comparative analysis of two independent groups was performed using the chi-squared test and the Mann–Whitney *U*-test. A value of *p* < 0.05 was accepted as statistically significant.

## Results

**Fig. 1:**
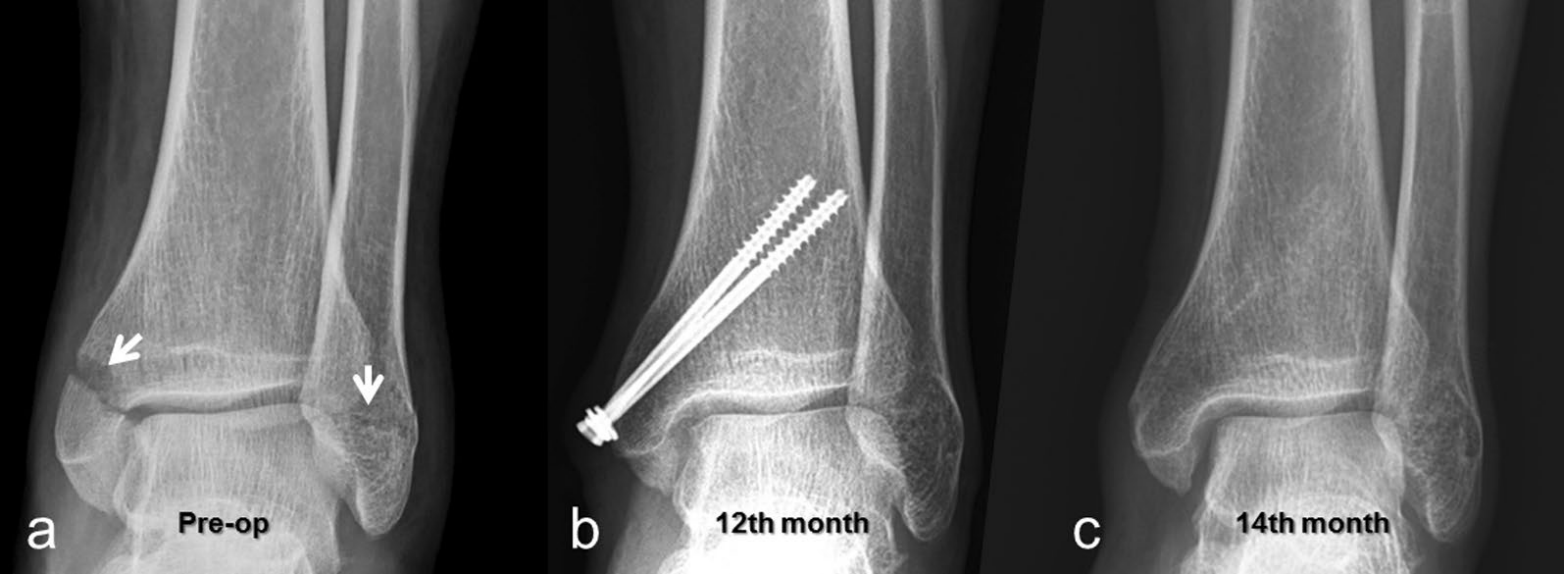
**a** Preoperative anteroposterior (AP) ankle radiograph of 44-year-old male patient with bimalleolar fracture (white arrows showing fractures); **b** At 12-month follow-up, fracture was united, but patient had pain over medial malleolus; **c** AP ankle radiograph 2 months after removal of screws

There were 23 patients in Group Mg (16 male, 7 female; mean age: 37.9 ± 17.7 years), and 25 patients in Group Ti (14 male, 11 female; mean age: 45.0 ± 15.7 years). Both groups were comparable concerning demographic and clinical characteristics. Age, sex, side, MM fracture type, associated fractures, and follow-up period were similar between groups (Table 1). At final follow-up examination, the AOFAS score was similar between groups. Fracture union was achieved in all MM fractures, but lateral malleolar nonunion was present in a patient in Group Ti. Grade of posttraumatic osteoarthritis according to KL was equally distributed in both groups. No deep infection or osteomyelitis was seen. Five patients in Group Ti underwent implant removal due to pain in three of them and difficulty in shoe-wearing in the other two. Two of these patients had isolated medial malleolar fractures, two bimalleolar fractures, and the other one trimalleolar fractures. Implant removal was performed after a mean time of 14.2 ± 3.1 months (12–19 months) (Table 2; Fig. 1).

**Table 1:** Demographic and clinical characteristics of patients

Variable	Group Mg (*n* = 23)	Group Ti (*n* = 25)	*p*-Value
Age (years ± SD)	37.9 ± 17.7	45.0 ± 15.7	0.110^a^
Sex (M/F)	16/7	14/11	0.252^b^
Side (R/L)	9/14	14/11	0.190^b^
Herscovici classification (*n*)			0.500^b^
Type B	12	12	
Type C	11	13	
Associated fractures (*n*)			0.470^b^
Isolated	13	10	
Lateral malleolus	6	9	
Lateral and posterior malleolus	2	5	
Posterior malleolus	2	1	
Follow-up (months)			0.869^a^
Mean months ± SD	24.7 ± 12.0	24.6 ± 9.1	
Range (min–max)	12–53	12–40	
Median	19	25	

*M* Male, *F* female, *R* right, *L* left, *Mg* magnesium, *Ti* titanium

^a^Mann–Whitney test

^b^chi-squared test

**Table 2:** Comparison of functional outcomes between groups

Variable	Group Mg (*n* = 23)	Group Ti (*n* = 25)	*p*-Value
AOFAS (score ± SD)	93.7 ± 8.8	90.0 ± 10.7	0.161^a^
OA grading			0.074^b^
Grade 0	21	17	
Grade 1	1	6	
Grade 2	0	2	
Grade 3	1	0	
Implant removal *n* (%)	0 (0%)	5 (20%)	0.031^b^

*AOFAS* American Orthopaedic Foot and Ankle Society, *OA* osteoarthritis, *Mg* magnesium, *Ti* titanium

^a^Mann–Whitney test

^b^chi-squared test

### Radiological findings of degradation of Mg screws

**Fig. 2:**
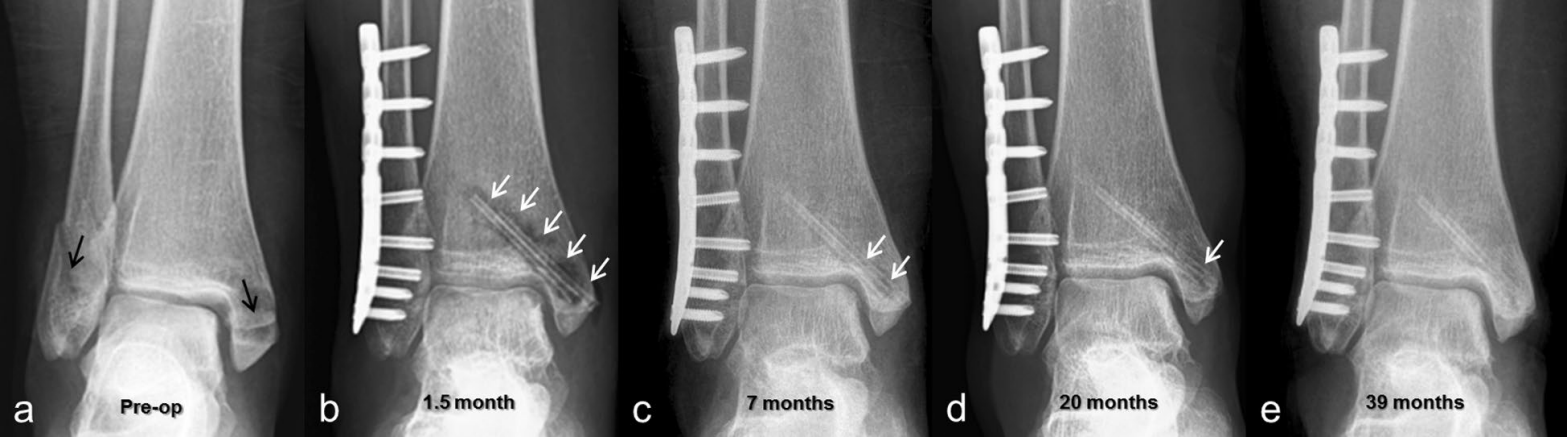
**a** Preoperative AP ankle radiograph of 45-year-old female patient with a bimalleolar fracture at initial admission (black arrows showing fractures); **b** Lateral malleolar fracture fixed with plate and screws and medial malleolar fracture fixed with two bioabsorbable Mg screws. At 1.5-month follow-up, gas shadows were observed around screws (white arrows); **c** At 7th month visit, fracture was united, and significant decrease in gas shadows was seen; **d** At 20th month follow-up, almost no gas shadows were visible; **e** At final follow-up, faded silhouette of screws was hardly noticeable

**Fig. 3:**
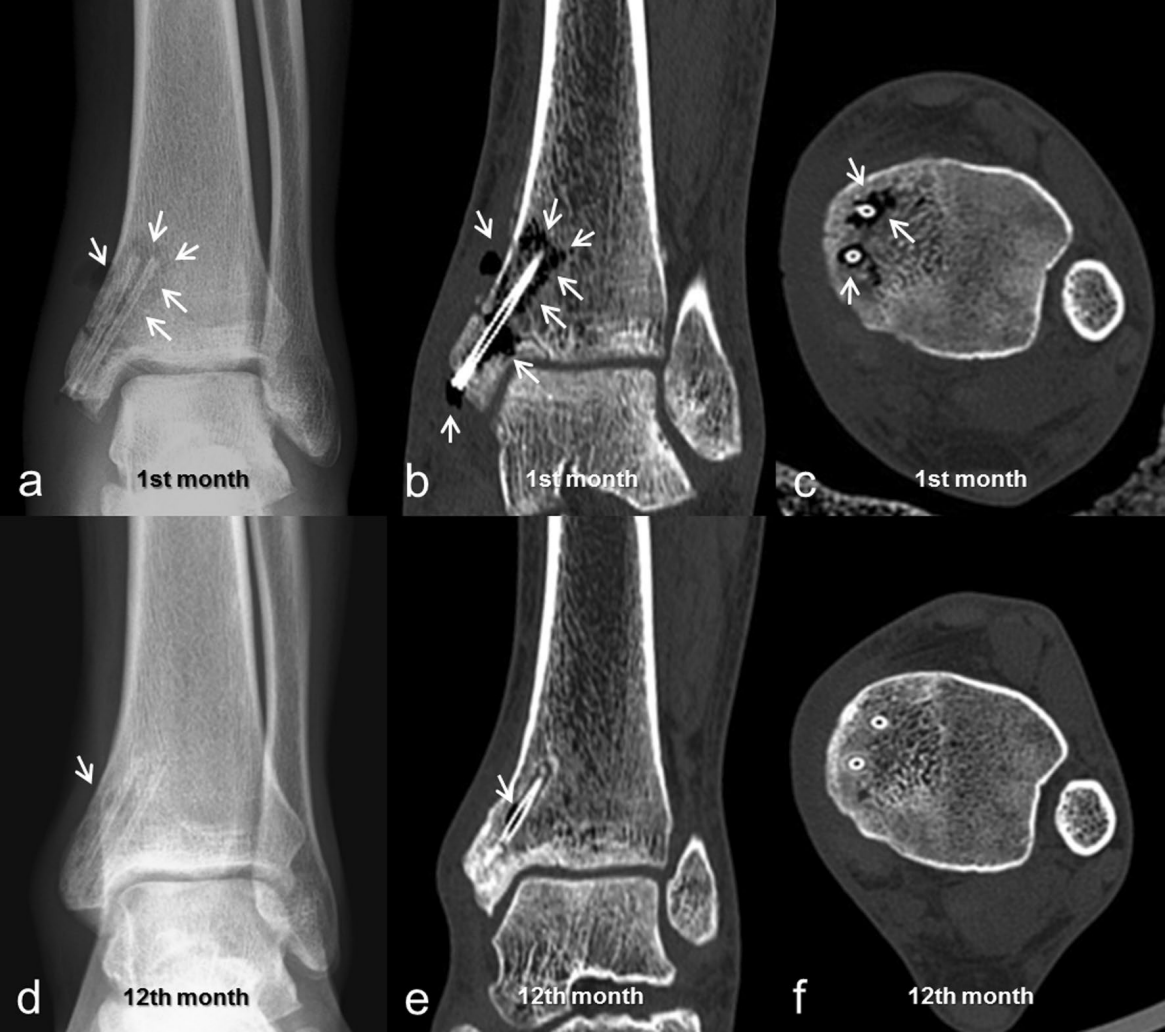
**a** Early postoperative ankle radiograph of 21-year-old male patient with isolated medial malleolar fracture. Fracture fixed with two bioabsorbable Mg screws. Note gas both in soft tissue and around screws (white arrows); **b** Coronal and **c** axial CT taken at first-month follow-up showed gas formation around screws and scattered throughout cancellous bone (white arrows); **d** AP ankle radiograph at first-year follow-up, almost no gas accumulations were seen. **d** Coronal and **e** axial CT taken at first-year follow-up confirmed almost total absorption of released gas

As the present work is a retrospective study, not all patients had scheduled radiographs taken at similar dates. However, all consecutive image files, CT scans and magnetic resonance imaging (MRI), were evaluated. All patients had gas in the soft tissue on early postoperative radiographs. Gas shadows were commonly observed within surgical incision plans and disappeared within 2–3 months. Later on, the gas formation was observed around the implants within the bone. The amount of gas increased in the first 4 months and began to decrease after the 6th month. At the end of the first year, almost no gas shadows were visible (Fig. 2). CT was performed in eight patients in Group Mg to evaluate union and fracture displacement at various time schedules. Similarly, gas was seen around the screws in early postoperative CT images, whereas later CT images showed the absorption of the gas (Fig. 3).

## Discussion

In this study, the fixation of MM fractures with two different implants was retrospectively compared. Clinical results at final follow-up were similar in both groups. Fracture union was achieved in all patients without major complications. Radiologically similar results were obtained regarding the development of posttraumatic osteoarthritis. However, the incidence of implant removal was statistically higher with titanium screws. Clinical and radiological outcomes in fixation of MM fractures with Mg screws were not inferior compared with titanium screw fixation, while this provided an important advantage as implant removal was prevented. Therefore, it can be argued that the use of bioabsorbable Mg implants is as safe and effective as conventional implants and does not require removal of the implant.

Initial clinical studies of bioabsorbable Mg implants were performed on hallux valgus deformity correction osteotomies [17, 22]. With the success of these studies, the indications were expanded to include various osteotomies and fracture fixation within the last decade [16, 18, 19, 23, 24]. However, few studies have reported the results of ankle fracture fixation [16, 18, 19]. Biber et al. [16] reported a successful outcome in an infrasyndesmotic lateral malleolar fracture. Similarly, Acar et al. [19] reported that they achieved union in another infrasyndesmotic lateral malleolar fracture. Kose et al. [18] evaluated short-term clinical and radiological results of MM fractures fixed with Mg screws in 11 patients. Excellent clinical results and fracture union without complications were obtained. However, no comparison has been made with titanium screws to date. The results of the current study are consistent with these previous studies. Bioabsorbable Mg screws provide adequate fixation for MM fractures. Furthermore, their use prevents implant removal operations.

Given that the soft tissue envelope around the ankle is thin, in particular around medial malleolus, implant removal is a frequent operation following ankle fractures. Rates of implant removal of up to 81% have been reported in current literature [6–9, 25, 26]. The most common symptoms are pain, prominent hardware, difficulty in shoe wear, and functional impairment. Many authors report that the reduction in patients’ complaints after implant removal is unpredictable, but some authors recommend routine removal [26]. However, these operations are prone to many complications such as broken or partially retained hardware, infection, compromised wound healing, and significant iatrogenic injuries [9, 27]. Kasai et al. [9] reviewed 80 ankle implant removal operations and reported an arterial injury in one patient, blistering in three, nerve injuries in three, skin necrosis in two, and infection in two. Besides, implant removal is a technically challenging surgical procedure that may require special equipment [27]. Therefore, these operations should not be underestimated. In this context, the prevention of implant removal operations provides significant benefits for both patients and surgeons. A few studies suggest the use of titanium headless compression screws to eliminate the need for implant removal in MM fractures. The authors argued that the headless screws did not cause implant-related irritation findings because the screws were fully countersunk into the bone [28–30]. Similarly, the headless design of Mg screws appears to be advantageous.

Bioabsorbable Mg implants are more expensive than conventional implants. Therefore, the initial surgical costs are high compared with titanium and steel implants. However, cost analysis studies in literature have shown that bioabsorbable implants are advantageous when total costs are calculated. Juutilainen et al. [31] performed a cumulative cost analysis for 140 patients who underwent ankle fracture treatment (92 bioabsorbable screw fixation versus 48 metallic screw fixation) and proposed that bioabsorbable screw fixation is more economical than metallic implants when all expenses (initial costs plus implant removal costs) are taken into account. In another study, Bostman et al. [32] reported that the financial advantage of bioabsorbable screws could only be obtained after a 21% implant removal rate in unimalleolar fractures. Klauser carried out a cumulative cost analysis based on the assumption of an 8% implant removal rate in hallux valgus osteotomy fixation and suggested that the use of bioabsorbable Mg screws in all hallux valgus treatment would save about €9 million in Germany [33]. Considering the high rate of implant removal, bioabsorbable screws may be cost effective in ankle fractures.

Bioabsorbable Mg screws are degraded by different mechanisms than polymer absorbable implants. Polymer implants are initially hydrolyzed and broken down into its monomers by enzymatic reaction, and later on, these monomers enter the citric acid cycle and are excreted from the body as water and carbon dioxide. During the enzymatic hydrolysis, the local pH decreases, and an acidic environment occurs around the implants [34]. In contrast, the degradation of Mg implants is an electrochemical reaction rather than enzymatic hydrolysis. After implantation of Mg screws, these react with the liquids in the tissue, especially water, and turn into Mg (OH)_2_ and hydrogen (H_2_) gas [12, 13, 15]. The resulting hydrogen gas is detected radiologically first in soft tissue and then in bone tissue. However, over time, this gas is absorbed, disappears from the environment, and cannot be detected with imaging. Furthermore, this reaction does not interfere with the fracture union [18]. Since hydrogen gas in the bone is observed as radiolucent sites, this may be considered to be osteolysis by unexperienced surgeons or radiologists using this implant for the first time.

In addition to favorable clinical results of bioabsorbable Mg screws, a few studies have also reported poor results and complications. Meier et al. [35] observed cyst formation in the early period and reported delayed union in five cases of acute scaphoid fractures. Radiographic cysts continued until the sixth month, but the clinical and radiological results of the patients were excellent after 1 year. These findings actually reflect the normal degradation process of Mg screws described above. In another case report, Mg screws were used in carpal arthrodesis surgery, and similarly, cyst formation and nonunion were reported [36]. Furthermore, Klauser reported the result of hallux valgus osteotomies fixed with Mg screws in a large series of patients composed of 100 cases. Three delayed wound healing, two deep surgical site infection, and one screw fracture were recorded [33]. Similarly, Windhagen et al. [22] reported problems with local wound healing in two patients who underwent hallux valgus surgery. Infection and delayed wound healing can develop due to a variety of factors and may not only be related to the implant itself. In the present study, no local wound healing problems or deep infection were observed.

There are some strengths and limitations to this study. First, the retrospective collection of data is the most critical limitation. Although ankle fractures are prevalent fractures, a relatively limited number of patients were included; however, the selection of patients treated with two compression screws for MM fracture decreased our number of patients. Besides isolated medial malleolar fractures, other patterns of ankle fractures were included, but both groups were statistically comparable in terms of several preoperative clinical and radiological features. All patients were followed up until fracture union was completed. The mean follow-up period was 2 years, which allowed us to evaluate the mid-term results including posttraumatic osteoarthritis. However, a longer follow-up will be required to detect long-term complications and sequelae.

In conclusion, bioabsorbable Mg and titanium screws have similar therapeutic efficacy in MM fracture fixation. Fracture union can be obtained with both screws without significant complications. Radiological outcomes were also similar. However, the prevention of implant removal operations with Mg screw fixation was the most crucial advantage. Due to this advantage, bioabsorbable Mg screws may be a favorable fixation option for this indication for the future.

## Data Availability

The datasets used and/or analyzed during the current study are available from the corresponding author on reasonable request.

## Abbreviations

MM: Medial malleolar
Mg: Magnesium
AOFAS: American Orthopaedic Foot and Ankle Society
KL: Kellgren–Lawrence
PLA: Poly-l-lactide
PGA: Polyglycolide
PDO: Polydioxanone
CT: Computed tomography
PACS: Picture archiving and communication system
MRI: Magnetic resonance imaging
